# Pain rather than self-reported sedentary time explains variation in perceived health and activity limitation in persons with rheumatoid arthritis: a cross sectional study in Sweden

**DOI:** 10.1007/s00296-016-3641-x

**Published:** 2017-01-25

**Authors:** Ingrid Demmelmaier, Pernilla Åsenlöf, Patrick Bergman, Birgitta Nordgren, Christina H. Opava

**Affiliations:** 1 Division of Physiotherapy, Department of Neurobiology, Care Sciences and Society, Karolinska Institutet, 23100, Alfred Nobels Allé 23, 141 83 Huddinge, Sweden; 2 Department of Neuroscience, Uppsala University, Uppsala, Sweden; 3 School of Education, Psychology and Sports Science, Linnaeus University, Kalmar, Sweden; 4 Department of Rheumatology, Karolinska University Hospital, Stockholm, Sweden

**Keywords:** Rheumatoid arthritis, Sedentary time, Sitting, Disability, Health, Multiple regression

## Abstract

To investigate (1) the amount of self-reported time spent sedentary among a large cohort of persons with rheumatoid arthritis (RA), and (2) the contribution of sedentary time to explain perceived health and activity limitation in RA beyond that of previously known correlates. This cross-sectional study used data from a postal questionnaire and the Swedish Rheumatology Quality registers (SRQ). The International Physical Activity Questionnaire was used to assess sedentary time (sitting) and moderate, vigorous and walking activity (MVPA). Sociodemographics, pain, fatigue, fear-avoidance beliefs, anxiety/depression, disease duration, MVPA and sedentary time were included in multiple regression models with perceived health (Visual Analogue Scale 0–100) and activity limitation (Stanford Health Assessment Questionnaire) as dependent variables. Results: In all 3152 (59%) of 5391 persons identified as eligible from the SRQ, responded to the questionnaire. 2819 individuals with complete data on all study variables were analysed. Mean time (SD) spent sedentary was 257 (213) minutes per day. Sedentary time did not contribute significantly to explain perceived health and only minimally to explain activity limitation. Instead, variation was mainly explained by pain; for perceived health (Beta = 0.780, *p* < 0.001) and for activity limitation (Beta = 0.445, *p* < 0.001).The results indicate a non-significant role of sedentary time and a need for increased focus on pain in the management of RA. Future studies should use prospective designs and objective assessment methods to further investigate the associations between sedentary time and health outcomes in persons with RA.

## Introduction

‘Sedentary time’ refers to the time spent in activities that do not increase energy expenditure substantially above the resting level (1.0–1.5 Metabolic Equivalent of Task, METs), such as watching computer screens or television, and is often measured by time spent in a sitting or reclining position [1, 2]. Evidence is accumulating on the negative effects of prolonged sedentary time, pointing to increased risk of cardiovascular disease, metabolic syndrome, and mortality in various populations [3–5]. Studies on physiological mechanisms indicate that inactivity and muscle disuse may cause low-grade chronic systemic inflammation [6], putting sedentary persons with inflammatory diseases such as rheumatoid arthritis (RA) at particular risk.

Impact and levels of sedentary time in populations with RA have been minimally explored. However, one study on patients with RA reported lower bone mass among those who spent prolonged periods of time sedentary compared to those who did not [7], and two recent accelerometer studies found persons with RA to spend on average almost 10 h per day sedentary [8, 9]. These figures are to be compared with studies conducted on the general population [10–13], reporting a mean daily sedentary time of 8–9 h, and on populations with long-term conditions such as stroke (13 h) [14] and osteoarthritis (10 h) [15].

Functioning is recognized as an important outcome in RA [16]. Based on the International Classification of Disability, Functioning and Health (ICF), the ICF core set for RA includes patient-reported outcomes that are used to complement medical measures [17]. Two crucial patient-reported outcomes, reflecting the over-arching ICF concepts of health and disability in RA, are perceived health and activity limitation [17].

Despite the accumulating evidence for the negative effects of prolonged sedentary time, its individual contribution to explaining health outcomes in RA is unknown. Previously identified predictors of health outcomes include pain, fatigue, depression, disease activity, radiographic progression [18, 19], physical activity [20], gender, and age [21]. The present study aimed to investigate (1) the amount of self-reported time spent sedentary among a large cohort of persons with RA, and (2) the contribution of self-reported sedentary time to explain perceived health and activity limitation in RA beyond that of a number of previously identified correlates.

## Methods

### Design and selection

We applied a cross-sectional design using the Swedish Rheumatology Quality Registers (SRQ) for selection. Criteria for inclusion were diagnosis of RA, an age of under 75 years, and a Stanford Health Assessment Questionnaire (HAQ) disability index score of ≤2. We approached 5391 individuals meeting the inclusion criteria of whom 3152 (58.5%) consented to participate. The selection procedure was the first step in the recruitment process for a physical activity intervention [22], thus motivating the inclusion criteria on age and disability index score. Participants were sent a letter containing information about the study along with the study questionnaire and provided their consent by returning the completed questionnaire. Non-responders were younger, had shorter disease duration, and were slightly more affected by their RA at their most recent medical examination as compared to responders [23]. Our study group comprised 2819 individuals with complete data on all study variables.

### Measures

#### Dependent variables

Perceived health was rated on a 100 mm visual analogue scale (VAS) from 0 (excellent health) to 100 (worst imaginable health), with high values indicating increased disease impact on health. The scale is considered valid and reliable in RA populations [24].

Activity limitation was assessed by the Stanford Health Assessment Questionnaire (HAQ) [25] with 20 items about daily activities performed during the past week, such as dressing and grooming, getting up, eating, taking care of personal hygiene, reaching, gripping, walking, and other common activities. Ratings were made on a four-point scale from ‘With no difficulty’ (=0) to ‘Unable to perform’ (=3). A total score between 0 and 3.0 was then calculated, where high values indicated increased activity limitation. The Swedish HAQ is considered valid and reliable in RA populations [26].

#### Independent variables

Background data on age, gender, education, income, number of adults in the household, pain (VAS 0–100), fatigue (VAS 0–100), fear-avoidance beliefs with a modified Fear-Avoidance Beliefs Questionnaire (0–24) [27], and anxiety/depression with one EuroQoL-5 Dimension (EQ-5D) [28] item (0–3) were collected via questionnaires. Data on disease duration were retrieved from the SRQ.

Moderate-to-vigorous physical activity (MVPA) was assessed by six items from the International Physical Activity Questionnaire (IPAQ) short form, which asked questions about the frequency and duration of vigorous-intensity, moderate-intensity, and walking physical activity, respectively. The responses were coded using established methods for the IPAQ [29]. Time spent in vigorous, moderate, and walking activity was weighted by the energy expended for these categories of activity to produce MET minutes of physical activity. The IPAQ has demonstrated acceptable test–retest reliability and criterion-related validity compared with accelerometers in the general population [30], and a mix of overestimation and underestimation of energy expenditure compared with accelerometers in an RA sample [31].

Sedentary time was assessed by the following item from the IPAQ short form: “During the past 7 days, how much time did you usually spend sitting on a weekday?” Responses were given in hours and minutes. The test–retest reliability of this item has been reported as good (Spearman rho values >0.7 in four country samples) and validity against accelerometers as acceptable [32].

### Data management and statistics

Respondents’ level of education was originally gathered in five categories: basic education, college, less than 2 years of university, three or more years of university, and other education. The two university education categories were subsequently collapsed into one, and the remaining alternatives into another. The number of adults in the household was initially recorded in four categories: living alone, living with one adult, living with two adults, and living with three or more adults. This was later modified to a simple yes/no answer as to whether there were other adults in the household. The variable of anxiety/depression was, due to very few study participants reporting extreme problems, dichotomized from three levels (no problems, some problems, and extreme problems) by collapsing the two last categories into one. The MVPA variable is the sum of the weighted MET minutes from the variables walking (the time reported times 3.3 MET), moderate physical activity (4 MET) and vigorous physical activity (8 MET).

The study sample is described using mean and standard deviation (SD) for continuous variables and by proportions for categorical variables. Independent sample *t* test and chi-square tests were used to compare the study sample with the participants that were excluded due to incomplete data.

The independent contributions of MVPA and sedentary time to the explanation of variation in activity limitation and perceived health, respectively, were analysed in a series of multiple linear regression models. The models were built by first entering the background variables (age, gender, education, income, number of adults in the household, anxiety/depression, pain, fatigue, fear-avoidance beliefs, and disease duration). Second, MVPA was entered and, in the final step, sedentary time. The variables were entered in this order to assess any additional contribution of sedentary time in addition to previously identified correlates. No signs of multicollinearity were observed; however, due to unsatisfactory residuals, the dependent variable ‘perceived health’ was transformed using the square root. The MVPA variable is the sum of the weighted MET minutes from the variables walking (the time reported × 3.3 MET), moderate physical activity (the time reported × 4 MET) and vigorous physical activity (the time reported × 8 MET).

All data were analysed using IBM Statistical Package for the Social Sciences (SPSS) for Windows version 21. Alpha levels were set to 0.05 for the multiple regression models and the individual contribution of each independent variable.

## Results

### Participants

The sample included 2819 individuals with complete data on all study variables. Descriptives and a comparison with participants excluded due to incomplete data are presented in Table 1.

**Table 1:** Descriptives of the study sample (*n* = 2819) compared to participants excluded due to incomplete data (*n* = 333)

	Study sample (*n* = 2819)	Excluded (*n* = 333)	*p*
Age, mean ± SD	59 ± 11	64 ± 10	<0.001
Gender, *n* (%)			0.480
Female	2059 (73)	250 (75)	
Male	760 (27)	84 (25)	
Education, *n* (%)			<0.001
University	953 (34)	72 (22)	
Not university	1866 (66)	262 (78)	
Income (below or above Swedish average), *n* (%)			<0.001
Below	1453 (51)	178 (73)	
Above	1366 (49)	65 (27)	
Other adults in household, *n* (%)			0.613
Yes	2137 (76)	249 (75)	
No	682 (24)	82 (25)	
Anxiety/depression (item 5 EQ-5D), *n* (%)			0.400
Yes	1006 (36)	127 (62)	
No	1813 (64)	207 (38)	
Pain (VAS 0–100)^a^, mean ± SD	30 ± 24	35 ± 27	0.001
Fatigue (VAS 0–100)^a^, mean ± SD	39 ± 26	42 ± 28	0.001
Fear-avoidance beliefs (mFABQ 0–24)^a^, mean ± SD	8 ± 6	8 ± 5	0.481
Disease duration (years), mean ± SD	4 ± 5	4 ± 3	0.532
MVPA (MET min/day), mean ± SD	2544 ± 2918	1735 ± 2615	<0.001
Sedentary time (min/day), mean ± SD	257 ± 213	160 ± 185	<0.001
Perceived health (VAS 0-100)^a^	30 + 24	34 + 27	0.007
Activity limitation (HAQ 0–3)	0.6 + 0.6	0.7 + 0.6	0.001

*EQ-5D* EuroQoL-5 Dimension, *VAS* Visual Analogue Scale, *MVPA* Moderate-to-Vigorous Physical Activity, *mFABQ* modified Fear-Avoidance Beliefs Questionnaire, *MET* Metabolic Equivalent of Task, *HAQ* Stanford Health Assessment Questionnaire

*p* values are based on independent sample *t* test and Chi-square-test

^a^High values indicates worse health and more fear-avoidance beliefs

Compared to individuals with incomplete data (*n* = 333), our sample was younger, had higher education and income, reported less pain and less fatigue, performed more MVPA, spent more time sedentary, had better perceived health and experienced less activity limitation.

### Sedentary time

Self-reported sedentary time was mean 257 (SD = 213) minutes per day.

### Contribution of sedentary time to explaining variation in perceived health and activity limitation

The outcomes of the regression models for explanations of perceived health and activity limitation are presented in Tables 2 and 3, respectively. The total amount of explained variance for each model is given, as well as the contribution of each independent variable.

**Table 2:** Outcome of the three multiple linear regression models with the square root of perceived health as the dependent variable (*n* = 2819)

Perceived health	Age	Gender	Level of education	Income	Number of adults in household	Anxiety/depression	Pain	Fatigue	Fear-avoidance beliefs	Disease duration	MVPA	Sedentary time	Coefficient of determination (*R* ^2^)
Model 1													0.741
Standardized beta	0.042	0.014	0.012	−0.018	−0.006	0.020	0.780	0.111	0.041	−0.006			
*p* value	<0.001	0.179	0.240	0.097	0.530	0.067	<0.001	<0.001	<0.001	0.563			
Model 2													0.742
Standardized beta	0.042	0.012	0.012	−0.018	−0.006	0.018	0.779	0.111	0.039	−0.006	−0.020		
*p* value	<0.001	0.256	0.241	0.111	0.519	0.084	<0.001	<0.001	<0.001	0.526	0.041		
Model 3													0.742
Standardized beta	0.043	0.013	0.010	−0.020	−0.006	0.018	0.780	0.111	0.039	−0.006	−0.019	0.017	
*p* value	<0.001	0.205	0.321	0.068	0.550	0.084	<0.001	<0.001	<0.001	0.521	0.053	0.092	

**Table 3:** Outcome of the three multiple linear regression models with activity limitation as the dependent variable (*n* = 2819)

Activity limitation	Age	Gender	Level of education	Income	Number of adults in household	Anxiety/depression	Pain	Fatigue	Fear-avoidance beliefs	Disease duration	MVPA	Sedentary time	Coefficient of determination (*R* ^2^)
Model 1													0.446
Standardized beta	0.145	0.126	0.001	−0.088	−0.001	0.039	0.448	0.145	0.130	0.064			
*p* value	<0.001	<0.001	0.962	<0.001	0.930	0.012	<0.001	<0.001	<0.001	<0.001			
Model 2													0.452
Standardized beta	0.145	0.118	0.001	−0.086	−0.002	0.035	0.445	0.144	0.125	0.062	−0.076		
*p* value	<0.001	< 0.001	0.967	<0.001	0.896	0.024	<0.001	<0.001	<0.001	<0.001	<0.001		
Model 3													0.454
Standardized beta	0.149	0.122	−0.004	−0.093	−0.001	0.035	0.445	0.144	0.125	0.062	−0.073	0.047	
*p* value	<0.001	<0.001	0.772	<0.001	0.967	0.024	<0.001	<0.001	<0.001	<0.001	<0.001	0.001	

## Discussion

This study provides new knowledge about the amount of time spent sedentary in a large sample of persons with RA, and demonstrated that self-reported sedentary time did not contribute substantially to explain the variation in perceived health and activity limitation. Rather, the major parts of variation were explained by self-reported pain, indicating that pain overrides the influence that sedentary time might have on both outcomes.

Spending approximately 4 h per day sedentary during waking hours is surprisingly little compared to results from other studies using the single IPAQ item on sitting. One study on general population samples from 20 countries reported sedentary time as median 5 h per day [10]. Another study on persons with multiple sclerosis (84% female, ambulatory and one-month relapse-free) reported a mean of 7.5 h/day [33], and a recent review found average levels falling between 8.5 and 9.6 h/day in older adults [34]. Our data did not provide any explanation for the low amount of sedentary time in the adult RA population, but this may be found in the operationalization of sedentary time as sitting time [35]. People living with RA may go earlier to bed at night due to fatigue, and remain longer in bed or even go back to bed due to morning stiffness and pain [36], leaving less time for daytime activities including sitting. Another explanation may be that spending much time sitting is not necessary during periods of remission [36], which may have been the case for some participants.

The explanatory value of time spent sedentary in our study was unexpectedly low, considering the amount of research reporting the negative impact of a sedentary lifestyle on health, as well as its contribution to the burden of chronic disease worldwide [37, 38]. Sedentary time has also been found to be negatively associated with functioning in osteoarthritis [15, 39]. The contribution of MVPA to explain variations in both perceived health and activity limitation in the present study was small. We had expected MVPA to make a greater contribution, as recent reviews have reported positive health outcomes of MVPA in RA [40], other rheumatic diseases [41] and osteoarthritis [39, 42]. However, it should be acknowledged that differences in design and methods, e.g. self-reports versus objective assessment of physical activity may explain part of the disparity in findings.

Pain was by far the strongest explanatory variable of variation for both outcomes in the present study, particularly regarding perceived health. The results thus indicate that pain overrides the influence of all other included independent variables, highlighting an urgent need to address pain in RA treatment and rehabilitation. Since pain is one cardinal symptom in RA, described as a combined effect of nociceptive signals during active joint inflammation flares and generalized, central pain mechanisms [43, 44], it is important to consider both mechanisms in RA management. Multidisciplinary programmes for pain rehabilitation have been developed to address the complex nature of long-term pain [45], but have not been fully implemented within rheumatology. Further research is needed to implement and evaluate multidisciplinary pain programmes, combining pharmacological and non-pharmacological interventions, adapted to the individual needs of each patient in RA populations [46].

### Methodological strengths and limitations

One strength of the study is the operational definition of sedentary time as a construct separate from physical activity, with these being scored as separate entities. Separating the two constructs into different behaviours is significant, as they may be determined by different bio-psycho-social factors. Different interventions will be needed depending on whether the aim is to decrease sedentary behaviours or to increase physical activity behaviours. Other strengths include the large and well-defined sample, made possible by the use of the SRQ for selection of participants.

Limitations refer to the use of self-reported data and its inherent risk of recall bias. Another limitation is the operational definition of sedentary behaviour as self-reported time spent sitting, and the fact that it does not capture all behaviours classified as sedentary, i.e. corresponding to an energy expenditure of <1.5 METs. There is thus a risk that the sitting time item from the IPAQ does not fully represent the actual sedentary time spent by the persons with RA included in the present study. In addition, studies comparing accelerometer and IPAQ data in persons with RA describe them to report less sedentary time and more high-intensity physical activity than when objectively assessed [47, 48]. Nevertheless, IPAQ was originally designed for population surveillances that allow for comparisons with other populations, and the sitting time item has acceptable measurement properties [32]. A combination of self-reports and accelerometers is recommended to reach high specificity in measurement [47, 48], but is unfortunately not yet feasible in epidemiological studies. Another limitation to our study is the lack of disease activity data, which were only available from the SRQ at a matching point in time for less than 50% of the study sample. It can thus not be excluded that disease activity contributes in explaining the assessed health outcomes, although this has not been the case in all studies on RA using multivariate approaches [49, 50]. The external validity of the study may be threatened by the small, but systematically skewed, data towards poorer health in the non-responders. Our results may, therefore, not be valid for an RA population with poorer health.

### Conclusions and implications for future studies

The present study is, to the best of our knowledge, the first large-sample study reporting sedentary time in adults with RA as separated from time spent physically active. Daily sedentary time was low and did not contribute to explaining the variation in perceived health and activity limitation. Instead, these outcomes were primarily explained by self-reported pain, indicating an increased focus on pain in the management of RA. Future prospective studies should test the hypothesis based on the current study, i.e. that sedentary time does not explain variation in perceived health and activity limitation in people with RA. Such studies may benefit from measurement refinements, e.g. the use of accelerometers combined with information on context, type, and duration of sedentary behaviours per se.
